# Clinical Characteristics of and Risk Factors for Fever after Endobronchial Ultrasound-Guided Transbronchial Needle Aspiration: A Retrospective Study Involving 6336 Patients

**DOI:** 10.3390/jcm9010152

**Published:** 2020-01-06

**Authors:** Kyoung Min Moon, Chang-Min Choi, Wonjun Ji, Jae Seung Lee, Sei Won Lee, Kyung-Wook Jo, Jin Woo Song, Jae Cheol Lee

**Affiliations:** 1 Department of Pulmonary, Allergy, and Critical Care Medicine, Gangneung Asan Hospital, College of Medicine, University of Ulsan, Gangneung 44608, Korea; pulmogicu@gmail.com; 2 Department of Pulmonary, and Critical Care Medicine, Asan Medical Center, College of Medicine, University of Ulsan, Seoul 100744, Korea; jack1097@naver.com (W.J.); jsdoc1186@daum.net (J.S.L.); iseiwon@gmail.com (S.W.L.); heathcliff6800@hanmail.net (K.-W.J.); jwsong@amc.seoul.kr (J.W.S.); 3 Department of Oncology, Asan Medical Center, College of Medicine, University of Ulsan, Seoul 100744, Korea; jclee@amc.seoul.kr

**Keywords:** endobronchial ultrasound-guided transbronchial needle aspiration, fever, incidence, risk factor

## Abstract

Endobronchial ultrasound-guided transbronchial needle aspiration (EBUS-TBNA) is a minimally invasive diagnostic for mediastinal and hilar lymphadenopathy/mass. This study investigated fever incidence and associated risk factors after EBUS-TBNA in 6336 patients who underwent EBUS-TBNA at Asan Medical Center from October 2008 to February 2018. Bronchoscopists evaluated participants’ medical records for fever the 24 h following EBUS-TBNA. Patients were placed in either a Fever group (*n* = 665) or a non-Fever group (*n* = 5671). Fever developed in 665 of 6336 patients (10.5%) with a mean peak body temperature of 38.3 °C (range, 37.8–40.6 °C). Multivariate analysis revealed that fever-associated risk factors after EBUS-TBNA are older age (adjusted OR 0.015, 95% CI (0.969–0.997), *p* = 0.015), bronchoscopic washing (adjusted OR 1.624, 95% CI (1.114–2.368), *p* = 0.012), more than four samples of EBUS-TBNA (adjusted OR 2.472, 95% CI (1.288–4.745), *p* = 0.007), hemoglobin levels before EBUS-TBNA (adjusted OR 0.876, 95% CI (0.822–0.933), *p* < 0.001), CRP levels before EBUS-TBNA (adjusted OR 1.115, 95% CI (1.075–1.157), *p* < 0.001), and a diagnosis of EBUS-TBNA tuberculosis (adjusted OR 3.409, 95% CI (1.870–6.217), *p* < 0.001). Clinicians should be aware of the possibility of fever after EBUS-TBNA because it is common. Additional, prospective, large-scale research should assess the need for prophylactic antibiotics for EBUS-TBNA.

## 1. Introduction

Endobronchial ultrasound-guided transbronchial needle aspiration (EBUS-TBNA) recently replaced mediastinoscopy for the determination of lymph node staging and diagnosis of thoracic diseases [1]. Real-time TBNA of mediastinal and hilar lymph nodes or masses under direct endobronchial ultrasonography guidance is safe with a good diagnostic rate [2,3]. EBUS-TBNA is mainly used for nodal staging in patients with suspected or established lung cancer [4]. The cumulative sensitivity of EBUS-TBNA in staging lymph nodes in patients with lung cancer ranged 85–100% [3,5,6,7]. This technique has also been performed in patients with sarcoidosis, tuberculosis, and lymphoma, and patients with unknown etiologic mediastinal lymphadenopathy [3,7,8,9]. Although complications associated with EBUS-TBNA are few, they do occur [3]; and as EBUS-TBNA use increases, significant and critical complications could increase also [1].

The major complications recently published in case reports are empyema, lung abscess, mediastinal abscess, intramural hematoma of the pulmonary artery, hemopneumo mediastinum, needle breakage, and pericarditis [10,11,12,13,14,15,16].

Incidence of fever after bronchoscopy has been reported to range from 5–14.2% [17,18,19,20,21], and the independent risk factors associated with a fever after bronchoscopy were identified as ultimate diagnosis of pulmonary tuberculosis and the severity of bleeding [18]. A few previous studies have investigated fever after EBUS-TBNA [22,23,24,25], and the incidence of fever after EBUS-TBNA was reported in the range of 0.03–20% [23,24,25]. However, the incidence in these studies was widespread, and the previous studies did not reveal any risk factors for fever after EBUS-TBNA. Therefore, in this study, we evaluated the incidence of fever and fever associated risk factors after EBUS-TBNA.

## 2. Materials and Methods

### 2.1. Definition and Inclusion Criteria

**Figure 1:**
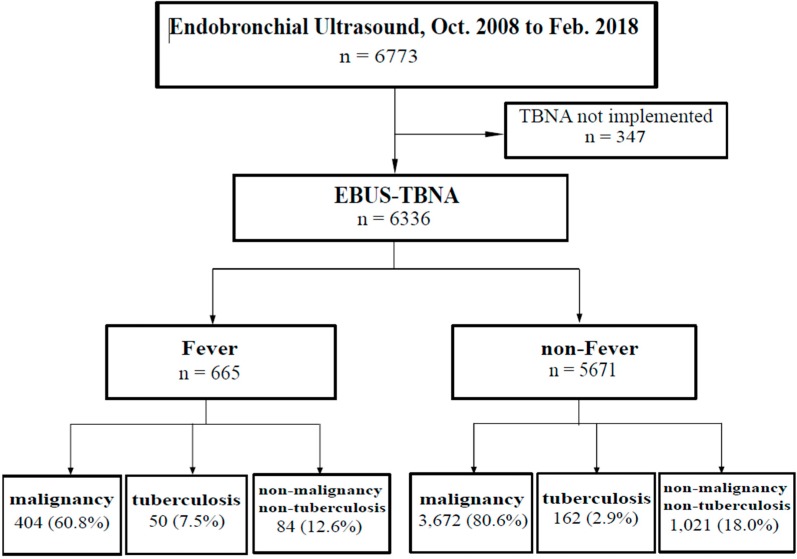
Study flowchart. EBUS-TBNA = endobronchial ultrasound-guided transbronchial needle aspiration.

This study was a single center, retrospective study conducted at Asan Medical Center, Seoul, South Korea. We selected patients who underwent EBUS-TBNA between October 2008 and February 2018 (Figure 1). The purpose of EBUS-TBNA was to assess a mediastinal staging or for tissue confirmation.

The subjects evaluated for fever by a physician every six to eight hours during the first 24 h following EBUS-TBNA; then were divided into two groups (Fever group or non-Fever group) according to presence or absence of febrile complication. Fever was defined as a temperature rise of axillary measurement point more than 37.8 °C. Patients who had a fever for 24 h prior to EBUS/EBUS-TBNA did not receive the procedure.

### 2.2. Patient Characteristics and Measure

Patient demographic characteristics and clinical data were retrieved from database and electronical medical records. Clinical manifestation included age, sex, fever, height, weight, and forced expiratory volume in one second (FEV1). Comorbidity included diabetes mellitus, hypertension, and hepatitis. Clinical data included smoking history, past medical history, sample number in EBUS-TBNA, diagnosis following EBUS-TBNA, additional procedures within 24 h of EBUS-TBNA, antibiotic use within three days of EBUS-TBNA, mortality (30, 60, and 90 days), and laboratory tests.

Additional procedures within 24 h of EBUS-TBNA included bronchoscopic washing, bronchoscopic biopsy, core needle biopsy, transbronchial lung biopsy, incisional biopsy, endoscopic ultrasound-guided fine-needle aspiration, core needle biopsy of the liver, surgical biopsy, and excisional biopsy. Antibiotics were classified as first-generation cephalosporin, third-generation cephalosporin, penicillin with beta-lactamase inhibitor, quinolone, macrolide, carbapenem, glycopeptide, and others. Laboratory tests included measurement of the values of C-reactive protein (CRP), white blood cell (WBC), hemoglobin, platelet, neutrophil, lymphocyte, eosinophil, monocyte, basophil, aspartate aminotransferase (AST), alanine aminotransferase (ALT), alkaline phosphatase (ALP), total bilirubin, gamma-glutamyl transferase (GGT), blood urea nitrogen (BUN), low density lipoprotein (LDL), and high density lipoprotein (HDL). Laboratory measurement were evaluated within one week prior to EBUS-TBNA.

The primary assessed outcome was the incidence of fever after EBUS-TBNA. Secondary assessed outcomes were risk factors associated with a fever after EBUS-TBNA.

### 2.3. EBUS-TBNA Procedure

An expert pulmonologist (physician who has experienced EBUS-TBNA for over 10 years in department of respiratory medicine) performed the EBUS-TBNA procedures. Patients were placed in a conscious sedated state with midazolam. Lidocaine was administered for local anesthesia. A standard conventional flexible bronchoscopy (model BF-T160 bronchoscope, Olympus, Tokyo, Japan) was initially used to examine the tracheobronchial tree. A linear array ultrasonic bronchoscope (BF-UC260FW; Olympus, Tokyo, Japan) with a dedicated 22-gauge needle (NA-201SX-4022; Olympus, Tokyo, Japan) was subsequently used to perform all needle aspiration procedure. Lymph node or mass confirmed by imaging were located by ultrasound. TBNA was performed two or three times to obtain sample using real-time ultrasonic needle guidance through 2.2 mm working channel in the bronchoscope. When necessary, doppler ultrasound was used to identify vessels. TBNA was performed from distant lymph node/mass to avoid needle contamination by sampling highly suspected of metastasis on chest CT or ^18^F-FDG-PET-CT.

### 2.4. Statistical Analysis

Categorical variables were analyzed using a chi-square test, and continuous variables were analyzed using Student’s t test. The odds ratios (OR) for the risk of fever after EBUS-TBNA were analyzed using a multivariate logistic regression model, including age, sex, any variables with a *p* < 0.20. A two-tailed *p*-value of less than 0.05 was considered statistically significant. The data were analyzed using SPSS statistic software (version 20.0; IBM Corp, Armonk, NY, USA) and R packages (The R Foundation, http://www.r-project.org/).

### 2.5. Ethics Approval

The Institutional Review Board of Asan Medical Center approved this study (IRB No. S2018-0440). The board waived informed consent because of the retrospective nature of the study. This study was performed according to the ethical standards of the Declaration of Helsinki, as revised in 2008.

## 3. Results

### 3.1. Patient Demographic and Clinical Characteristics

**Figure 2:**
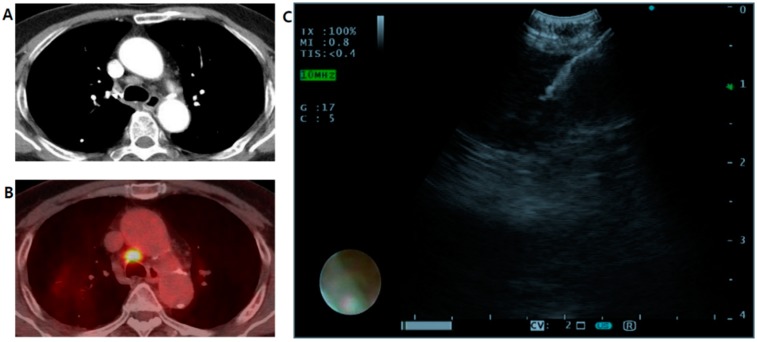
Example of a patient with a mediastinal lymph node diagnosed by EBUS-TBNA. (**A**,**B**) Enhanced chest CT and PET-CT show an enlarged hypermetabolic lymph node in the right lower paratracheal area (SUV max = 9.9). (**C**) Real-time TBNA of an enlarged lymph node (station 4R) under direct endobronchial ultrasonography guidance. EBUS-TBNA = endobronchial ultrasound-guided transbronchial needle aspiration; SUV max = maximum standardized uptake value.

Of the 6773 cases who underwent EBUS, 6336 were included in this study except for 347 who did not undergo EBUS-TBNA (Figure 1 and Figure 2). The incidence of fever after EBUS-TBNA was 665 cases (10.5%). At the initial measurement, fever occurred in four out of 665 patients in the Fever group (0.006%). The mean peak body temperature was 38.3 °C (range, 37.8–40.6 °C). The median age of these 6336 cases was 65.0 years (range, 14–96 years). The male-to-female ratio was 68:32. The mean FEV1 was 80.28% (range, 18–150%). The comorbidities included diabetes mellitus (4745 cases, 74.9%), hypertension (2282 cases, 36.0%), and hepatitis (203 cases, 3.2%). Smoking history included ‘never smoker’ (2310 cases, 36.5%), ‘ex-smoker’ (3049 cases, 48.1%), and ‘current smoker’ (783 cases, 12.4%). The past medical history included tuberculosis (277 cases, 4.4%), and malignancy (4945 cases, 78.0%). The 12,389 lymph nodes/mass were biopsied with EBUS-TBNA, malignancy was identified in 4992 (40.3%) and non-malignancy in 6188 (50.5%) (not presented).

### 3.2. Associations of Fever after EBUS-TBNA with Clinical and Laboratory Findings

Table 1 summarizes the clinical characteristics of the Fever group and the non-Fever group. There were significant differences between the two groups in mean age and age groups (all, *p* < 0.001): (10 y ≤ age < 40 y) and (70 y ≤ age < 100 y) had a higher rate in the Fever group than in the non-Fever group, and (40 y ≤ age < 70 y) had a lower rate in the Fever group than in the non-Fever group (Table S1). There were no significant differences between the two groups in sex, body mass index, FEV1, and smoking history (all, *p* > 0.05). A past medical history of tuberculosis and the 90 day mortality in the Fever group were significantly higher than in the non-Fever (*p* < 0.001 and *p* = 0.002, respectively). There was no significant difference between the two groups in a past medical history of malignancy (*p* = 0.620). However, there was a significant difference in the past medical history of lung cancer (*p* = 0.002) (Table S1).

The mean EBUS-TBNA samples in the Fever group was significantly higher than in the non-Fever group (2.14 for the Fever group vs. 2.03 for the non-Fever group, *p* = 0.004). More than four EBUS-TBNA samples in Fever group was a significantly higher rate than in the non-Fever group (5.7% for Fever group vs. 3.8 for the non-Fever group, *p* = 0.021) (Table 2). An EBUS-TBNA tuberculosis diagnosis in the Fever group was significantly higher than in the non-Fever group (7.5% for Fever group vs. 2.9% for the non-Fever group, *p* < 0.001) (Figure 1 and Table 2).

Differences in additional procedures within 24 h of EBUS-TBNA between the two groups are summarized in Table 3. Bronchoscopic washing, bronchoscopic biopsy, and transbronchial lung biopsy in the Fever group were significantly higher rate than in the non-Fever group (*p* < 0.001, *p* = 0.030, and *p* = 0.039, respectively). The mean number of additional procedures in the Fever group were significantly higher than in the non-Fever group (1.99 for the Fever group vs. 1.84 for the non-Fever group, *p* < 0.001). Only EBUS-TBNA in the Fever group was significantly lower than in the non-Fever group (29.8% for the Fever group vs. 38.4% for the non-Fever group, *p* < 0.001). However, each of the ≥1 and ≥2 categories of additional procedures in the Fever group were significantly higher than in the non-Fever group (70.2% for the Fever group vs. 61,1% for the non-Fever group, *p* < 0.001; 25.9% for the Fever group vs. 21.3% for the non-Fever group, *p* = 0.008, respectively) (Table 3).

CRP, WBC, platelet, neutrophil, ALT, ALP, GGT, and creatinine before EBUS-TBNA in the Fever group were significantly higher than in the non-Fever group (all, *p* < 0.001). Hemoglobin, lymphocyte, protein, calcium, phosphorus, sodium, chloride, and cholesterol before EBUS-TBNA in the Fever group were significantly higher than in the non-Fever group (*p* < 0.001, *p* < 0.001, *p* = 0.026, *p* = 0.038, *p* < 0.001, *p* < 0.001, *p* < 0.001, and *p* < 0.001, respectively) (Table S2).

### 3.3. Risk Factors Associated with Fever after EBUS-TBNA

Table 4 shows that the ORs of fever after EBUS-TBNA for age, bronchoscopic washing, more than four samples of EBUS-TBNA, hemoglobin, and C-reactive protein before EBUS-TBNA, and tuberculosis diagnosis following EBUS-TBNA. When we assessed the factors associated with a fever after EBUS-TBNA using a multivariate analysis, fever after EBUS-TBNA was significantly associated with age (adjusted OR 0.015, 95% CI (0.969–0.997), *p* = 0.015), bronchoscopic washing (adjusted OR 1.624, 95% CI (1.114–2.368), *p* = 0.012), more than four samples of EBUS-TBNA (adjusted OR 2.472, 95% CI (1.288–4.745), *p* = 0.007), hemoglobin before EBUS-TBNA (adjusted OR 0.876, 95% CI (0.822–0.933), *p* < 0.001), CRP before EBUS-TBNA (adjusted OR 1.115, 95% CI (1.075–1.157), *p* < 0.001), and tuberculosis diagnosis following EBUS-TBNA (adjusted OR 3.409, 95% CI (1.870–6.217), *p* < 0.001) (Table 4).

**Figure 3:**
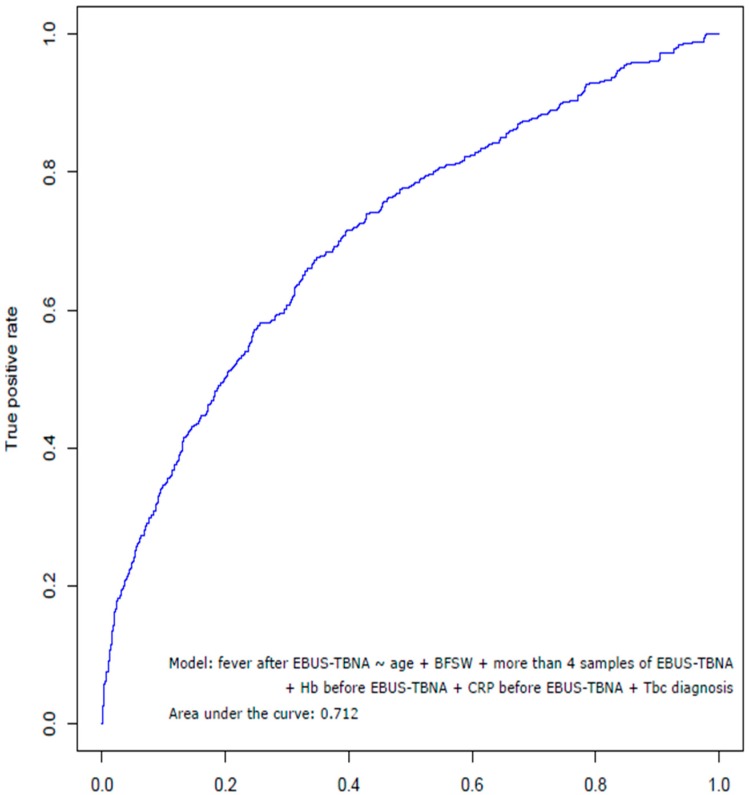
Receiver operating characteristic curve showing the statistical performance of the factors associated with the febrile complication after EBUS-TBNA. EBUS-TBNA = endobronchial ultrasound-guided transbronchial needle aspiration; BFSW = bronchoscopic washing; Hb = hemoglobin; CRP = C-reactive protein; Tbc = tuberculosis.

We used receiver operating characteristic (ROC) curves to assess the statistical performance of the models for risk factors associated with a fever after EBUS-TBNA. The area under ROC was 0.712 (Figure 3).

## 4. Discussion and Conclusions

Fever after EBUS-TBNA was 10.5% (665 patients of 6336) in our study. A few previous studies have reported the incidence of fever after EBUS-TBNA. Kim et al. [23] reported the fever developed in 110 cases (19.9%) in retrospective single-center study of 552 South Korean patients. On the other hand, Asano et al. [24] and Caglayan et al. [25] reported that fever developed in only four cases (0.05%) and one case (0.03%) in a retrospective multi-center study of 7345 Japanese patients and 3123 Turkish patients, respectively. Since these two studies were questionnaire surveys [24,25], recall bias was potentially high. Generally, patients may be hesitant to report complications, therefore underestimating the incidence of fever after EBUS-TBNA.

To the best of our knowledge, this is the first study for revealing the risk factors related to fever after EBUS-TBNA. In a retrospective study of 552 patients who underwent EBUS-TBNA, Kim et al. [23] did not reveal any risk factors after analysis by multiple logistic regression, though there were significant differences in the number of sampled lymph node or mass, and the final diagnosis of tuberculosis between the fever group (110 patients) and the non-fever group (442 patients). In previous survey studies of 7345 Japanese patients [24] and 3123 Turkish patients [25], there were no analyses of risk factors associated with a fever after EBUS-TBNA. However, unlike previous studies [23,24,25], the risk factors related to fever after EBUS-TBNA were assessed after analysis of multiple logistic regression: age, bronchoscopic washing, more than four samples of EBUS-TBNA, hemoglobin, CRP, and tuberculosis of EBUS-TBNA diagnosis (Table 4).

Although the cause of fever after EBUS-TBNA is unclear, transient bacteremia may be one of the possibilities of fever. Previous studies reported that transient bacteremia caused by oropharyngeal bacteria after EBUS-TBNA is a possible cause of fever after EBUS-TBNA [10,11]. Haas et al. [11] showed that the puncture site of EBUS-TBNA is necrotic or cystic, resulting in a decrease in bacterial attachment clearance due to low blood flow and reduced resistance. Steinfort et al. [26] support that contaminated TBNA needles with oropharyngeal flora may cause focal infection at the puncture site of TBNA. Therefore, identifying the exact location of the EBUS-TBNA needle tip, by ultrasound imaging, during the perforation is thought to help prevent fever after EBUS-TBNA.

It is important to disinfect the device according to the instructions [27] and to observe the post-procedure process for signs of infection carefully. Until now, routine diagnostic bronchoscopy does not recommend prophylactic antibiotics except for patients who are asplenic, have a heart valve prosthesis, or a previous history of endocarditis [28]. Takagi et al. [29] reported that antibiotic prophylaxis in EBUS-TBNA does not affect body temperature and laboratory test related inflammation. However, severe complications, such as mediastinal abscess can have fatal consequences [13,14,16,30]. Although rare in our study, there were five cases of mediastinal abscess among in 6336 patients with EBUS-TBNA. In addition, because EBUS-TBNA is more widely used in recent years, reports of complications associated with its use have increased [11,14,22,24,30,31]. Therefore, for patients with risk factors associated with a fever after EBUS-TBNA antimicrobial agents against oral and nasopharyngeal organisms may be helpful based on this study, if used empirically [10]. In the present study, there were 72 cases of antibiotic use before EBUS-TBNA for prophylactic. Using antibiotics before EBUS-TBNA in the Fever group was significantly more frequent than in the non-Fever group (5.7% for the Fever group vs. 0.6% for the non-Fever group, *p* < 0.001). There was significant difference in the case of receiving prophylactic antibiotics in the Fever group: bronchoscopic washing (*p* = 0.001), age (*p* = 0.044), hemoglobin before EBUS-TBNA (*p* < 0.001), and CRP before EBUS-TBNA (*p* < 0.001) (not presented). Antibiotics with the highest percentages between the Fever group and the non-Fever group were third-generation cephalosporin (28.9%), and penicillin with beta-lactamase inhibitors (29.4%), respectively (Table S3). Further research is needed to assess the need for prophylactic antibiotics and to identify when such prophylaxis is needed.

The limitation of this study is that it is retrospective. This allow data selection bias and underestimation of fever after EBUS-TBNA. However, it is unlikely that the overall incidence of fever after EBUS-TBNA would vary significantly because this is the largest study so far using real-world data except for survey studies [24,25]. The reason for the 90 day mortality of the Fever-group is higher than the non-Fever group is not clear.

In summary, EBUS-TBNA is a safe method but occasionally can cause mild to severe complications. Fever after EBUS-TBNA is common, but in most cases, it is temporary and does not cause major problems. However, as use of EBUS-TBNA increases worldwide, more cases of fever after EBUS-TBNA should be expected. Thus, clinicians should be aware of the possibility of fever after EBUS-TBNA and the significantly associated factors: older age, bronchoscopic washing, more than four EBUS-TBNA samples, low hemoglobin levels, high CRP levels, and EBUS-TBNA diagnosed tuberculosis. In the future, prospective large-scale studies with severe complications (including mediastinal abscess) and all medications (including prophylactic antibiotics) are required to confirm the relationship between fever and EBUS-TBNA.

## Figures and Tables

**Table 1:** Characteristics of patients between the Fever group and the non-Fever group.

Characteristics	Fever (N = 665)	Non-Fever (N = 5671)	*p*-Value
Clinical manifestation			
Age, year, median (range)	65.0 (14–96)	66.0 (14–90)	
Sex, male, *n* (%)	456 (68.6)	3,868 (68.2)	0.848 ^1^
Height, cm	162.1 ± 12.8	162.4 ± 11.8	0.547 ^2^
Weight, kg	61.2 ± 23.0	62.5 ± 15.3	0.051 ^2^
Body mass index, kg/m^2^	23.3 ± 10.4	23.8 ± 12.3	0.297 ^2^
FEV1, %	76.9 ± 17.9	80.6 ± 18.5	0.127 ^2^
Comorbidity, *n* (%)			
Hypertension	245 (36.8)	2037 (35.9)	0.477 ^1^
Diabetes mellitus	64 (9.6)	509 (9.0)	0.581 ^1^
Hepatitis	24 (3.6)	179 (3.2)	0.682 ^1^
Smoking History, *n* (%)			0.477 ^1^
Never smoker	237 (35.6)	2073 (36.6)	
Ex-smoker	336 (50.5)	2713 (47.8)	
pack-year	35.4 ± 24.1	35.0 ± 23.6	0.763 ^2^
Current smoker	72 (10.8)	711 (12.5)	
pack-year	32.2 ± 20.9	36.1 ± 23.8	0.183 ^2^
Past medical history, *n* (%)			
Malignancy	514 (77.3)	4,431 (78.1)	0.620 ^1^
Tuberculosis	52 (7.8)	225 (4.0)	**<0.001 ^1^**
Mortality, *n* (%)			
30 days mortality	14 (2.1)	88 (1.6)	0.283 ^1^
60 days mortality	30 (4.5)	185 (3.3)	0.092 ^1^
90 days mortality	52 (7.8)	280 (4.9)	**0.002 ^1^**
Day from EBUS to death, median (range)	51.5 (3–89)	47.0 (3–90)	

EBUS-TBNA = endobronchial ultrasound-guided transbronchial needle aspiration; FEV1 = forced expiratory volume in one second. ^1^ Chi-squared test; ^2^ Student’s *t*-test; *p* < 0.05 is shown in bold.

**Table 2:** Sample number and ultimate diagnosis of EBUS-TBNA between the Fever group and the non-Fever group.

Parameters	Fever (N = 665)	Non-Fever (N = 5671)	*p*-Value
Sample number of EBUS-TBNA, mean	2.14	2.03	**0.004 ^2^**
≥2, *n* (%)	424 (63.8)	3599 (63.5)	0.898 ^1^
≥3, *n* (%)	172 (25.9)	1372 (24.2)	0.340 ^1^
≥4, *n* (%)	38 (5.7)	214 (3.8)	**0.021 ^1^**
5, *n* (%)	1 (0.2)	21 (0.4)	0.723 ^1^
Final diagnosis of EBUS-TBNA, *n* (%)			
Malignancy	404 (60.8)	3672 (64.8)	**0.042**
Tuberculosis	50 (7.5)	162 (2.9)	**<0.001**
Non-malignancy & Non-tuberculosis	84 (12.6)	1021 (18.0)	**0.001**
Follow-up	1 (0.2)	10 (0.2)	1.000
Loss of follow-up	1 (0.2)	0	0.105

EBUS-TBNA = endobronchial ultrasound-guided transbronchial needle aspiration. ^1^ Chi-squared test; ^2^ Student’s *t*-test; *p* < 0.05 is shown in bold.

**Table 3:** Additional procedures within 24 h from the day of EBUS-TBNA for the Fever group and the non-Fever group.

Parameters	Fever (N = 665)	Non-Fever (N = 5671)	*p*-Value
Additional procedure, *n* (%)			
Bronchoscopic washing	419 (63.0)	3080 (54.3)	**<0.001 ^1^**
Bronchoscopic biopsy	140 (21.1)	1000 (17.6)	**0.030 ^1^**
Core needle biopsy	40 (6.0)	287 (5.1)	0.293 ^1^
Transbronchial lung biopsy	10 (1.5)	42 (0.7)	**0.039 ^1^**
Number of additional procedures, mean	1.99	1.84	**<0.001 ^2^**
Only EBUS-TBNA, *n* (%)	198 (29.8)	2180 (38.4)	**<0.001 ^1^**
≥1 type, *n* (%)	467 (70.2)	3491 (61.1)	**<0.001 ^1^**
≥2 types, *n* (%)	172 (25.9)	1208 (21.3)	**0.008 ^1^**
≥3 types, *n* (%)	16 (2.4)	84 (1.5)	0.097 ^1^
4 types, *n* (%)	1 (0.2)	7 (0.1)	0.588 ^1^

EBUS-TBNA = endobronchial ultrasound-guided transbronchial needle aspiration. ^1^ Chi-squared test; ^2^ Student’s *t*-test; *p* < 0.05 is shown in bold.

**Table 4:** Risk factors associated with the febrile complication in patients with EBUS-TBNA.

	Univariate Analysis		Multivariate Analysis	
	*p*-Value	Crude OR (95% CI)	*p*-Value	Adjusted OR (95% CI)
Age	0.202	0.996 (0.989–1.002)	**0.015**	0.983 (0.969–0.997)
Sex	0.848	1.017 (0.855–1.209)	NA	NA
Body mass index	0.317	0.991 (0.975–1.008)	NA	NA
Bronchoscopic washing	**<0.001**	1.433 (1.214–1.691)	**0.012**	1.624 (1.114–2.368)
More than four samples of EBUS-TBNA	**0.016**	1.545 (1.084–2.204)	**0.007**	2.472 (1.288–4.745)
Hemoglobin ^1^	**<0.001**	0.750 (0.715–0.786)	**<0.001**	0.876 (0.822–0.933)
C-reactive protein ^1^	**<0.001**	1.150 (1.128–1.173)	**<0.001**	1.115 (1.075–1.157)
White blood cells ^1^	**<0.001**	1.104 (1.080–1.128)	NA	NA
Platelets ^1^	**<0.001**	1.003 (1.002–1.004)	NA	NA
ALP ^1^	**<0.001**	1.002 (1.001–1.003)	NA	NA
GGT ^1^	**0.009**	1.002 (1.000–1.003)	NA	NA
Cholesterol ^1^	**<0.001**	0.991 (0.989–0.993)	NA	NA
Tuberculosis of EBUS-TBNA diagnosis	**<0.001**	2.765 (1.992–3.837)	**<0.001**	3.409 (1.870–6.217)

OR = odds ratio; CI = confidence interval; ALT = alanine aminotransferase; ALP = alkaline phosphatase; GGT = gamma-glutamyltransferase; EBUS-TBNA = endobronchial ultrasound-guided transbronchial needle aspiration. ^1^ Values before EBUS-TBNA; *p* < 0.05 is shown in bold.

## Supplementary Materials

The following are available online at https://www.mdpi.com/2077-0383/9/1/152/s1, Table S1: Subset of patient characteristics between the Fever group and the non-Fever group; Table S2: Comparison of laboratory findings before and after EBUS-TBNA between the Fever group and the non-Fever group; Table S3: The use of antibiotics within 3 days from the day of EBUS-TBNA.
