# Supplementary material for: Clinical Characteristics of and Risk Factors for Fever after Endobronchial Ultrasound-Guided Transbronchial Needle Aspiration: A Retrospective Study Involving 6336 Patients
Source: J Clin Med. 2020 Jan 6;9(1):152. doi: 10.3390/jcm9010152 (PMC7019550; doi:10.3390/jcm9010152)
Supplement: Supplementary file 1 [file jcm-09-00152-s001.pdf]

Supplementary Table 1. Subset of patient characteristics between the Fever group and the non-Fever group

**Characteristics**	**Fever (N=665)**	**non-Fever (N=5671)**	***P*-value**
Age, mean (year)	63.2	63.8	**<0.001**[1]
Age group, *n* (%)			**<0.001**[2]
10 (10y ≤ age < 20y)	6 (0.9)	10 (0.2)	
20 (20y ≤ age < 30y)	19 (2.9)	42 (0.7)	
30 (30y ≤ age < 40y)	28 (4.2)	153 (2.7)	
40 (40y ≤ age < 50y)	40 (6.0)	420 (7.4)	
50 (50y ≤ age < 60y)	112 (16.8)	1173 (20.7)	
60 (60y ≤ age < 70y)	211 (31.7)	1880 (33.2)	
70 (70y ≤ age < 80y)	211 (31.7)	1721 (30.3)	
80 (80y ≤ age < 90y)	37 (5.6)	269 (4.7)	
90 (90y ≤ age < 100y)	1 (0.2)	3 (0.1)	
Past medical history of malignancy, *n* (%)			
Lung	408 (76.7)	3,391 (74.2)	**0.002**[2]
Gastrointestinal	33 (6.2)	319 (7.0)	
Hematologic	19 (3.6)	54 (1.2)	
Head and neck	14 (2.6)	109 (2.4)	
Breast	12 (2.3)	126 (2.8)	
HBP	11 (2.1)	142 (3.1)	
Esophageal	10 (1.9)	122 (2.7)	
Urologic	6 (1.1)	117 (2.6)	
Brain	3 (0.6)	42 (0.9)	
Gynecologic	2 (0.4)	35 (0.8)	
Others[†]	5 (0.9)	45 (1.0	
Unknown-origin	9 (1.7)	67 (1.5)	
Past medical history of tuberculosis, *n* (%)			
Pulmonary	36 (69.2)	165 (73.3)	**0.002**[2]

Lymphadenitis	8 (15.4)	38 (16.9)
Miliary	3 (5.8)	0
Gastrointestinal	1 (1.9)	1 (0.4)
Pleurisy	1 (1.9)	13 (5.8)
Cardiac	1 (1.9)	3 (1.3)
Others‡	2 (3.8)	2 (0.9)
Unspecified	0	3 (1.3)

HBP = hepatobiliary and pancreatic. †This included musculoskeletal, skin, thymus, and cardiac malignancy.

‡This included spinal and endobronchial tuberculosis, and tuberculous meningitis.

[1]Student`s t-test; [2]Chi-squared test; $P < 0.05$ is shown in bold.

Supplementary Table 2. Comparison of laboratory findings before and after EBUS-TBNA between the Fever group and the non-Fever group

Laboratory	Before EBUS-TBNA			After EBUS-TBNA		
	Fever (N=665)	non-Fever (N=5671)	*P*-value (t-test)	Fever (N = 665)	non-Fever (N = 5671)	*P*-value (t-test)
CRP, mg/dL	4.4±5.6	1.6±3.1	**<0.001**	6.5±5.8	4.1±5.6	**<0.001**
WBC, 10^3/μL	8.8±4.3	7.5±2.9	**<0.001**	10.0±4.6	8.5±3.9	**<0.001**
Hemoglobin, g/dL	12.1±1.8	13.0±1.7	**<0.001**	11.6±1.9	12.7±1.7	**<0.001**
Platelet, 10^3/μL	286.7±102.2	262.1±86.2	**<0.001**	286.5±112.1	261.6±93.6	**<0.001**
Neutrophil, /μL	6018.5±3864.9	4715.1±2586.1	**<0.001**	7292.2±4066.3	5704.0±3486.2	**<0.001**
Lymphocyte, /μL	1798.2±688.6	1950.4±750.0	**<0.001**	1677.4±723.0	1857.8±868.2	**<0.001**
Eosinophil, /μL	224.8±283.9	221.3±337.2	0.804	229.9±292.6	225.8±314.4	0.785
Monocyte, /μL	15.5±64.3	19.1±79.0	0.203	800.4±421.9	636.2±363.6	**<0.001**
Basophil, /μL	32.7±27.8	31.7±22.5	0.352	33.5±30.8	31.5±30.4	0.191
Protein, d/dL	6.8±0.7	6.9±0.6	**0.026**	6.8±0.8	6.9±0.7	**0.001**
AST, IU/L	25.6±21.3	24.5±18.0	0.190	28.0±23.6	26.1±26.3	0.082
ALT, IU/L	23.4±23.1	22.1±24.7	**<0.001**	25.5±26.2	23.9±26.3	**<0.001**
ALP, IU/L	99.3±80.4	86.3±59.3	**<0.001**	104.8±91.8	89.9±72.1	**<0.001**
T-Bil, mg/dL	0.5±0.5	0.5±0.5	0.328	0.6±0.5	0.6±0.6	0.607
GGT, IU/L	59.3±98.1	43.4±77.1	**0.016**	75.9±77.7	65.5±96.9	0.261
Uric acid, mg/dL	4.7±1.7	5.0±1.5	0.322	4.5±2.1	5.0±1.8	0.439
BUN, mg/dL	15.1±8.2	15.1±6.2	**0.001**	14.6±9.0	15.6±7.6	**<0.001**
Creatinine, mg/dL	1.0±1.1	0.9±0.6	**0.033**	1.0±1.1	0.9±0.7	0.181
Calcium, mg/dL	9.0±0.7	9.1±0.6	**0.038**	8.8±0.6	8.9±0.6	**0.011**
Phosphorus, mg/dL	3.5±0.7	3.6±0.6	**<0.001**	3.3±0.8	3.5±0.7	**<0.001**
Sodium, mEq/L	138.3±3.5	139.2±3.5	**<0.001**	137.0±3.7	138.2±3.9	**<0.001**
Potassium, mEq/L	4.3±0.5	4.3±0.4	0.365	4.2±0.4	4.3±0.4	**<0.001**
Chloride, mEq/L	101.0±3.9	102.3±3.7	**<0.001**	100.5±4.1	102.0±4.1	**<0.001**
Cholesterol, mg/dL	156.6±36.8	168.4±36.9	**<0.001**	149.8±39.8	165.1±39.6	**<0.001**

LDL, mg/dL	114.2±33.1	110.1±31.4	0.476	107.5±40.8	109.3±33.0	0.806
HDL, mg/dL	42.3±14.6	46.6±14.8	0.095	41.6±14.3	43.2±14.3	0.562
Triglyceride, mg/dL	112.0±72.2	119.2±62.5	0.494	125.2±67.7	135.0±82.0	0.506

CRP = C-Reactive Protein; WBC = White Blood Cell; AST = Aspartate Aminotransferase; ALT = Alanine Aminotransferase; ALP = Alkaline Phosphatase; T-Bil = Total Bilirubin; GGT = Gamma-glutamyltransferase; BUN = Blood Urea Nitrogen; LDL = Low Density Lipoprotein; HDL = High Density Lipoprotein; $P < 0.05$ is shown in bold.

Supplementary Table 3. The use of antibiotics within 3 days from the day of EBUS-TBNA

**Antibiotics**					**Fever (N=665)**	**non-Fever (N=5671)**	***P*-value**
**Antibiotics only before/after EBUS-TBNA, n (%)**					0	0	
**Use of antibiotics, n (%)**					38 (5.7)	34 (0.6)	**<0.001[1]**
**Antibiotics before EBUS-TBNA**							
Start from the day of EBUS-TBNA, day					1.63	2.24	0.322[2]
Duration, day					3.42	4.35	0.179[2]
Classification, *n* (%)							0.049[1]
1stCS					0	3 (8.8)	
	3rdCS				11 (28.9)	4 (11.8)	
		PN+BI			10 (26.3)	10 (29.4)	
			QN		1(2.6)	7 (20.6)	
	3rdCS			ML	2 (5.3)	1 (2.9)	
		PN+BI	QN		5 (13.2)	4 (11.8)	
		PN+BI		ML	3 (7.9)	0	
Others[†]					6 (15.8)	5 (14.7)	
**Antibiotics after EBUS-TBNA**							
Start after the day of EBUS-TBNA, day					1.66	1.91	0.176[2]
Duration, day					5.68	3.68	0.127[2]
Classification, *n* (%)							0.043[1]
3rdCS					0	6 (17.6)	
	QN				5 (13.2)	7 (20.6)	
		PN+BI			10 (26.3)	8 (23.5)	
			CBP		3 (7.9)	1 (2.9)	
	QN	PN+BI			6 (15.8)	2 (5.9)	
			CBP	GP	6 (15.8)	1 (2.9)	
Others[‡]					8 (21.1)	9 (26.5)	

EBUS-TBNA = endobronchial ultrasound-guided transbronchial needle aspiration; 1stCS = first generation

cephalosporin; 3^{rd}CS = third generation cephalosporin; PN = penicillin; BI = beta-lactamase inhibitor; QN = quinolone; ML = macrolide; CBP = carbapenem; GP = glycopeptide.

†This included ML, incosamide, GP, 3^{rd}CS with 1^{st}CS, 3^{rd}CS with incosamide, 4^{th}CS with incosamide, QN with incosamide, GP with 3^{rd}CS, GP with QN, and CBP with aminoglycoside.

‡This included ML, 1^{st}CS, 4^{th}CS, GP, CBP with PN, 3^{rd}CS with ML, 3^{rd}CS with QN, 3^{rd}CS with incosamide, CBP with QN, GP with PN, 1^{st}CS with aminoglycoside, 2^{nd}CS with aminoglycoside, and QN with aminoglycoside.

[1]Chi-squared test; [2]Student`s t-test; $P < 0.05$ is shown in bold.
